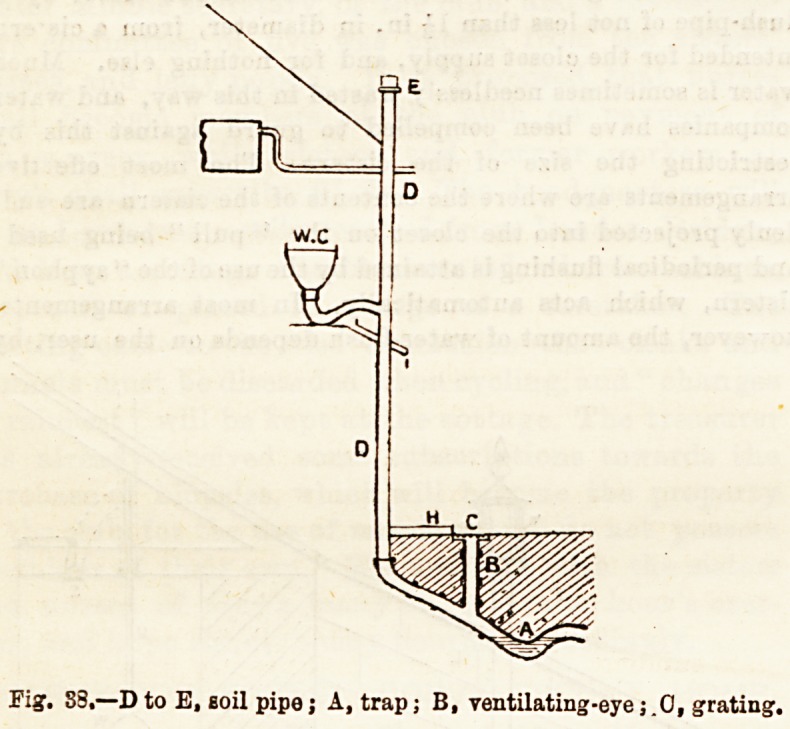# "The Hospital" Nursing Mirror

**Published:** 1896-09-12

**Authors:** 


### "The Hospital" Nursing Mirror.

Being the Extra Nursing Supplement of "The Hospital" Newspaper.

[Contributions for this Supplement should be addressed to the Editor, The Hospital, 428, Strand, London, W.C., and should have the word "Nursing" plainly written in left-hand top corner of the envelope.]

### News from the Nursing World.

### THE LONDON HOSPITAL NURSING HOME.

The addition to the Nursing Home at the London Hospital is almost completed, and is admirable in every way. A special feature of the new building will be a spacious sick-room on the ground floor to accommodate ten beds. The bedrooms are a very good size, and the furniture chosen, of light polished wood, is charming, and all-sufficient, each room being supplied with a large hanging cupboard, in three divisions, fitted in cornerwise from floor to ceiling so as to economise space and give a maximum of accommodation; a table, a chest of drawers, wash-hand-stand with cupboard, beds of most comfortable dimensions, chair and clothes basket. The distempering of the walls will be in pretty colours, and the paint white, so that the rooms will be as bright and cheerful as possible. Linoleum will cover the floors, as it does now in all the bedrooms in the present home, the general effect being warmer and more snug than polished boards, the latter also meaning a greater expenditure of domestic labour. The comfort of the London Nursing Home impresses a visitor more with each visit, and it is good to see the consequent pride taken by the nurses in their little sanctums. When the new building is ready for occupation, which will not be for a few weeks yet, this largest of our metropolitan voluntary hospitals may with justice congratulate itself on possessing better nurses' quarters than any other in London. We are glad to take this opportunity of stating, in reference to the article on the London Hospital Training School, in last week's "Mirror," that recently the sisters' hours off duty have been extended. They now have every other Sunday free from nine a.m. to eleven p.m., in addition to two half days a month from two to ten p.m., with "theatre pass" if desired. In the same article, in speaking of the night nurses' sleeping accommodation, the word "cubicle" is used in error. There are no cubicles in the building; all nurses and probationers have a separate bedrooms.

### WANDSWORTH AND CLAPHAM INFIRMARY.

At the last meeting of the Wandsworth and Clapham Board of Guardians the Relief Committee, which it will be remembered was appointed to inquire into the general condition of the nurses at the infirmary, submitted a report, and after some discussion the proposal to build a new nurses' home was agreed to, for which plans are to be invited forthwith. On Dr. Neal's report that the nurses needed more than a fortnight's holiday in the year, it was agreed to allow a month's leave in the future, and to at once increase the staff. This is setting to work in the right way to improve matters, and the Board are to be congratulated on their decision. We trust that the question of allowing the nurses a half-hour for breakfast, which has been referred to the Infirmary Committee, will meet with due consideration, and that this extension of time will be authorised.

### DISTRICT NURSING IN NORTH ARGYLL.

Now is the happy moment for sales of work and entertainments in aid of provincial charities, when the remotest parts of the country swarm with visitors from London and other great cities. Lady Victoria Campbell and the Hon. Helen Gardyne have seized the opportunity for a two-days' sale in aid of the District Nursing Fund of the Island of Tiree (the first ever held in the island by the way), at the Hall, Scarinish. There was a good display of all kinds of fancy articles, and of work done by ladies in Tiree and Mull. A number of people were present, and the promoters of the sale were well satisfied with the results.

### FIR VALE INFIRMARY, SHEFFIELD.

Miss Thomlinson, the lady superintendent of the Fir Vale Infirmary, is giving up her present post, which she has held for seven years past, to be married. A pleasant little ceremony took place the other day, when the resident medical officers and nurses presented Miss Thomlinson with a handsome case of silver-backed brushes and mirror as a wedding gift, and a token of sincere regard and esteem. Dr. Sorley made the presentation on behalf of the nurses, expressing the regret felt by the staff at losing one who had endeared herself to them all by her unfailing kindness and tact.

### A NURSES' HOME FOR SALISBURY INFIRMARY.

The annual meeting of the governors of Salisbury Infirmary was held on August 27th, during the course of which it was decided, on the recommendation of the treasurer, Mr. W. Pinckney, cordially seconded by the president, Lord Pembroke, that the building of a home for the nursing staff should be at once proceeded with. At present the nurses are very inadequately housed in the hospital building, and proper and up-to-date accommodation is badly needed. Plans were prepared some time ago, but their carrying out was postponed for lack of funds. Now, owing to a recent legacy, Mr. Pinckney thought the building committee should be requested to go on with the needful work for the accommodation of their "capable and hard-working nurses." Some people might object to the proposal on the ground of decreasing subscriptions, but he thought he might confidently predict, after thirty-seven years' experience of the infirmary, that when the public saw the need of help they would come forward with the required assistance. Lord Pembroke made some wise remarks upon the necessity of providing the nurses with a place to go to for rest and quiet when not on duty, and added his conviction that with better quarters the efficiency of the staff would increase. It is very satisfactory to note that everywhere throughout the country the necessity and justice of providing the nurses in our public hospitals with fitting and comfortable accommodation is being recognised, and we heartily congratulate the authorities of the Salisbury Infirmary on their decision. We hope the building will be proceeded with as promptly as possible.

### GUY'S NURSES ON WHEELS.

The nursing staff of Guy's Hospital are not going to be left behind in the matter of bicycling. A meeting was held on September 1st in the Matron's house to discuss no less a project than the inauguration of a cycle club for the sisters and nurses of the hospital. A unanimous assent was given to the proposal. Miss Nott-Bower was elected treasurer, and Sister Minnie secretary, of the club, and a representative committee of five was chosen from the sisters, nurses, and lady pupils. It is proposed that nurses shall become members of the club on payment of 10s. a year, while sisters and former workers at Guy's shall subscribe £1 1s. The headquarters will be at Lewisham, easily reached from London Bridge; and here a cottage has been rented, where machines, &c., will be placed in charge of a caretaker. The meeting came to the wise conclusion that cloaks and bonnets must be discarded when cycling, and "changes of raiment" will be kept at the cottage. The treasurer has already received some subscriptions towards the purchase of bicycles, which will become the property of the club, for the use of members who do not possess machines of their own. We cordially wish the sisters and nurses of Guy's many an enjoyable hour's exercise, and hope the club may flourish exceedingly.

### CHARNWOOD FOREST CONVALESCENT HOME.

A pleasant "At Home" was held at the Convalescent Home, Charnwood Forest, the other day. Unfortunately, the weather was somewhat rainy, but the guests, after visiting the various rooms, were treated to an enjoyable concert in the hall. The home is situated in charming country, well sheltered from cold winds, and its beds are in much request. Mr. A. W. M. Burder is its hon. secretary, and Miss Napier is the matron.

### NURSING AND POTATOES.

The Durham Board of Guardians recently had occasion to advertise for a nurse. Only one reply resulted, this coming from a young person who could put forward no other qualification than experience in the potato and fruit business. This is a sample of the difficulties Guardians have to encounter in obtaining nurses for union infirmaries.

### BRISTOL GENERAL HOSPITAL.

The nurses of the Bristol General Hospital spent a very pleasant evening on August 25th, when the annual distribution of prizes took place. By the kind invitation of the chairman, Mr. Joseph Storrs Fry, a number of friends interested in the work of the institution were present. Letters from the examiners were read testifying to the excellence of the papers sent in by the prize winners, and, more important still, to the good practical work in the wards during the past year. The following is the list of the successful candidates: In Anatomy and Physiology (First year nurses): K. Hunt, first prize; A Jones, second prize; A. Jenks, third prize; (Second year nurses): F. C. Davis, first prize; L. Culverwell, second prize; L. Griffiths, third prize. In Medical and Surgical Nursing (First year nurses): K. Hunt, first prize; A. Jones and N. Stow were equal for the second prize; (Second year nurses): F. L. Peake, first prize; F. C. Davis, second prize; L. Griffiths, third prize. The new wing to the Nurses' Home is now finished, and is to be opened on the 28th of the present month. This will give twenty additional rooms and a sitting-room for the staff, making now in all fifty-six bed-rooms and two sitting-rooms for the use of the nurses.

### THE STAFFORDSHIRE INSTITUTION FOR NURSES.

The twenty-fourth annual report of the Staffordshire Institution for Nurses is very satisfactory reading, recording as it does a year "of continued prosperity and progress." The staff, including district nurses and probationers, numbers 120, and the earnings reached £4,6[illegible]6 2s. 4d. A sum of £234 15s. was divided among them last February in bonuses. The committee strongly urge the necessity for their making provision for old age and sickness as far as possible, and, thus encouraged, fifty have taken out policies in the Royal National Pension Fund for Nurses, and others are preparing to follow this wise example. Many of these nurses received invitations to Marlborough House last summer, and eight of them were able to enjoy the pleasure of being present on that occasion, accompanied by their lady superintendent. Another Staffordshire nurse was a proud member of the deputation of twelve who, on March 12th of this year, presented the Pension Fund Nurse's wedding gift to the Princess Maud of Wales. It is pleasant to note the cordial appreciation of Miss Shirley's services as lady superintendent with which the report concludes.

### PROMOTION IN PROVINCIAL HOSPITALS.

In last week's *Nursing Supplement* a "Provincial Nurse" complained of the system of promotion at one "of the largest provincial hospitals," apparently with some reason, for it is certainly hard lines for the nurses trained in its wards that in six years two only should have been promoted to the post of sister, while there were eighteen vacancies during that time. It is surely a mistake, however, to look at the matter from the point of view only of the disappointed nurses. The infusion of new blood, so to speak, is very necessary now and again, for the world of a country hospital is a limited one, and things are apt to fall into grooves if no outside influences or fresh ideas and ways are introduced. It is undoubtedly for the good of the institution that vacancies on the permanent staff shall sometimes be filled by nurses from other hospitals, though if harmony and contentment are to reign this must be done judiciously and in proper proportion.

### SHORT ITEMS.

The sixth annual report of the Ladies' Nursing Association, Jarrow, states that last year Nurse McLeary made in all 3,509 visits.—The sale of work lately held at the District Home, Wolverhampton, in aid of the Queen Victoria Nursing Institution, realised, after deducting all expenses, a sum of £82 10s.—A successful garden party was given the other day by Mrs. Taylor, of Ashdown, Apperley Bridge, to the subscribers and friends of the Apperley and Greengates Nursing Fund. The number of subscriptions to this fund has largely increased during the past year.

### Hygiene: For Nurses.

By JOHN GLAISTER, M.D., F.F.P.S.G., D.P.H.Camb., Professor of Forensic Medicine and Public Health, St. Mungo's College, Glasgow, &c.

### XXIII. —SANITARY FITTINGS (*continued*) — DIFFERENT FORMS OF CLOSETS—THE SOIL-PIPE.

THE hopper closet is mainly found in two forms, viz., the long conical and the short hopper. The former is decidedly objectionable, by reason of it becoming easily fouled, and being imperfectly flushed. Less objection can be offered to the short form, and provided that the water-flush is adequate, it is an efficient fitting. Another form, known as the "pedestal" hopper, by Jennings, and which is flushed by siphon action, is also very efficient.

The "flush" closet embraces several forms, which are

Fig. 36.—BUCHAN'S "CARMICHAEL" CLOSET.
A, the receiver; B, the trap; G, the exit pipe; N, ventilating pipe P, water-flush; F, floor of apartment.

often made of one piece of earthenware, composing both closet and trap, and which may be made of the plainest or most ornamental character With the addition of a "hinged" seat, they may also be used as slop closets, as they have no valves or other mechanism liable to be put out of gear. Another merit is that they can be fitted into position without any surrounding woodwork. Pedestal in shape, they are divisible into two kinds, viz., the "wash-out" and the "wash-down" forms. They are so differentiated by reason of the primary point of lodgement of the dejecta; in the former it is a slightly projecting ledge, in the latter it is the trap itself. In the former class, therefore, much of the force of the water-flush is spent in dislodging the contents of the basin, and there is a liability for the contents to be swept into the trap only, and not through it. This objection does not apply to the wash-down form. Fig. 36 represents a good form of wash-down closet.

While these general points of difference are pointed out, it must be said that between the best types of either form of closet there is not much to choose in respect of efficiency. Of all the types mentioned, preference, however, must be given to the wash-down closet, formed of one piece of earthenware and fixed in position without covering of any kind. Another form of closet, once used but now generally condemned, is the "trapless" closet, so called because the water in the basin was intended to serve the double purpose of receiving the dejecta and acting as a trap. Should the valve by which the water is retained in the basin be, from any cause, shifted from its seat, there is nothing to interpose between the atmosphere of the soil pipe and the house interior. Deservedly, therefore, this closet has been condemned, and is now out of use.

Among the many makers of these fittings of the best class are Doulton, Twyford, Jennings, Shanks and Co., and others.

Whatever the form of closet in use, however, none can remain clean without periodical scrubbing with strong soda solution, after the use of which some effective disinfectant fluid should be used, when the trap, with proper flushing, will keep in good order for a week.

The characteristics of a good closet may be summed up in the following points, viz.: (1) It should be of a form to enable perfect dislodgement of the contents by the water-flush; (2) this dislodgement should be sudden and complete; (3) it should possess a sufficient trap; (4) all its parts should be easily cleansed; and (5) the water-flush should be ample.

THE WATER-FLUSH.—The amount of water to be used at one act of flushing should never be less than two, and, when possible, three gallons, and it should reach the closet by a flush-pipe of not less than $1\frac{1}{2}$ in. in diameter, from a cistern intended for the closet supply, and for nothing else. Much water is sometimes needlessly wasted in this way, and water companies have been compelled to guard against this by restricting the size of the cistern. The most effective arrangements are where the contents of the cistern are suddenly projected into the closet on the "pull" being used; and periodical flushing is attained by the use of the "syphon" cistern, which acts automatically. In most arrangements, however, the amount of water-flush depends on the user, by

Fig. 37.—E to J, soil pipe; C, trap; D, ventilating opening; G, grating on surface; F, cleansing-eye of trap; A, branch pipe from closet M; M, a valve closet, the trap of which, however, is not shown.

the length of time in which the "pull" is held. Carelessness in this respect has much to do with the inefficiency of any fitting.

The best type of cistern for closet-flushing ought to combine the following features, viz.: (1) To supply at each time of action three gallons of water; (2) to fill rapidly after being emptied; (3) to project the water suddenly and forcibly into the closet; and (4) to supply the same quantity of water at each action, irrespective of the user of the fitting.

The junction of the earthenware closet and the branch pipe to soil pipe—which is usually composed of lead—is one which demands the greatest care on the part of a workman (1) because any breach in the joining material exposes the closet-room to the air of the soil pipe, and (2) owing to the different expansibilities by heat of the substances connected. Common uniting materials such as red lead and others, for the above reasons, are apt to be imperfect, or to become so. This difficulty has quite recently been overcome by the new metallo-keramic joint, patented by Doulton. This forms a perfect connection, and from experiments we have made with it would appear to be a "fast" connection.

The Soil Pipe.—The main pipe used for carrying off dejecta

Fig. 38.—D to E, soil pipe; A, trap; B, ventilating-eye; C, grating.

is called the soil pipe. It is usually composed of lead or iron, and is jointed at intervals by rounded, flanged, or "filled" joints. It ought always to be placed on the outside of an outer wall of the house. The various fittings for the reception of excretory products are united to it by branch pipes, and each fitting is trapped from the branch pipe, and therefore from the soil pipe. Fig. 37 demonstrates the soil pipe passing down the exterior of the house-wall and receiving the branch connection from the closet M.

The soil pipe should extend from a point at least three feet above the house-eaves to a point below the level of the ground, where it is joined to an intercepting ventilating trap, which disconnects it from the house-drain. The diameter of the soil pipe ought to be the same throughout its entire length, and right angles ought to be avoided, else ventilation will be interfered with. The opening of the soil pipe should never be located near attic windows. Inspection of the soil pipe ought to be periodically made, as all jointed pipes are apt to "shake," especially when exposed on the sunny side of the house, on account of the expansion and contraction which the metal undergoes.

The size of the soil pipe must be regulated by the amount of work it is expected to do, and has therefore direct relation to the number of fittings connected with it. For an ordinary house of two or three flats a four-inch diameter pipe will amply suffice; for larger premises, such as hotels, &c., a five or six-inch pipe. (Fig. 38.)

The lead of soil pipes is attacked by the gases dissolved in the water which passes through them and by gases of decomposition. Corrosion often ends in perforation. When this takes place the house-interior is exposed to the atmosphere of the soil pipe, and the inmates, to consequent harmful effects. If the soil pipe be composed of iron, careful inspection of the joints with the lead branch pipes is necessary on account of the galvanic action which is liable to be set up between these metals.

### Novelties for Nurses.

### AN OPPORTUNITY IN BOOTS AND SHOES.

The London Shoe Company's Sale.

Many of us fought our way into the premises of the London Shoe Company last season on sale day, and those of us who survived have still some wonderful purchases with which to astonish our friends who either feared the crowd too much or had not known in time to go. Unlike many London sales, it was a perfectly genuine one, and not on the lines of the "stock-taking" and "damaged by fire and water"—sales so dear to the heart of improvident woman. On September 21st, and the three following days, an inauguration sale will be held at the company's new City warehouse, 123 and 125, Queen Victoria Street, City. The object of the sale is merely to induce customers to visit the splendid new premises, which are on so vast a scale that a crush would hardly be possible. All the ordinary stock will be offered with one-fifth off the usual price, and country orders reaching the City warehouse during the four sale days will be charged at sale prices; but it is no use ordering beforehand. Just now the weather leads one to dreams of reliable soles and waterpoof, or at least blacking, leather "uppers" rather than to glacé or French kid, but after having purchased a pair of the company's strong country boots at 19s. 9d., and triumphantly deducted the one-fifth, we that are wise will turn our thoughts to brighter days in store, and provide ourselves from among the alluring goods displayed. The "Haseldene," a walking shoe in patent and calf, is hard to beat, as it contrives to combine comfort with a smart toe, a rare combination. Another very nice walking shoe at a lower price is in glacé kid, either button or lace, which can be had with smart, medium, or square toes. Garden parties are over for this year, or the "Cromwell" walking shoes in glacé with cut steel buckles would have been much in demand, but they are not too thick for daily wear indoors and doubtless will commend themselves to many during the sale. So many members of the nursing world are regular customers of the L. S. Company that special attention has been paid to their needs. The limitations of uniform give but small opportunities for personal adornment, and boots and shoes are among the few items of her toilette in which a hospital nurse is allowed an individual choice. For wear "on duty" the company provides very comfortable noiseless slippers. They are made with straps over the instep and can be had in morocco or glacé. A dainty shoe for a tired foot is offered in softly-tinted cloth trimmed with fur, the heel being of moderate height. Unquestionably the most suitable and prettiest cycling boot for ladies is that designed by the L. S. Company. It has, however, already had such a vogue this season that I need merely say it is a combination of shoe with patent sole and long gaiter—a gaiter, moreover, which fits and does not deform the foot. A shoe will shortly appear made on the same principle, only without the gaiter. We must not forget to mention that about 2,000 pairs of coloured satin shoes are to be cleared off at one shilling per pair, and these are of really good material and well shaped.

### Wants and Workers.

[The attention of correspondents is directed to the fact that "Helps in Sickness and to Health" (Scientific Press, 428, Strand) will enable them promptly to find the most suitable accommodation for difficult or special cases.]

A lady desires to find a post for an experienced nurse as attendant on an invalid, where she will have no heavy lifting. Excellently recommended. Reply Mrs. H., care of the Editor, 428, Strand, W.C.

A lady is anxious to hear of a home for invalid nurses within easy reach of London, not seaside, and in a bracing air, where every comfort and care would be taken of a nurse requiring rest.

Nurse Mary will have a vacancy in October for a nurse wishing to spend her holiday at her pleasant house in Cheshire. Applications, with stamped envelope for reply, should be addressed to her at 57 Harders Road, Peckham, London.

### Nurses in 1896—Their Quarters, Hours, and Food.

### ST. BARTHOLOMEW'S HOSPITAL.

### I.—TERMS OF TRAINING.

AN agreement for four years is signed by the ordinary probationers at St. Bartholomew's Hospital, that is to say, their training extends over three years, at the expiration of which they receive a certificate, if satisfactory, and serve as staff nurses for the remainder of the time. A preliminary examination on simple subjects is exacted from the probationers. Examinations play an important part in the curriculum at St. Bartholomew's. At the end of the first year's training they are required to pass an examination "in such matters as they have had an opportunity of becoming acquainted with since entering the hospital." If this examination is passed satisfactorily, and the candidates prove themselves otherwise efficient, they are employed as "staff probationers" for the rest of the three years. At the expiration of this time their knowledge is again tested by examination. During the second and third years they receive regular instruction in medical and surgical nursing from members of the hospital staff. At the end of the third year a certificate of competency is presented to those who, besides giving satisfaction in ward work, have passed both examinations creditably, and they are appointed staff nurses. With regard to the preliminary examination which candidates are required to pass, besides satisfying the examiners that they possess general intelligence and have received a fair education, they must have an elementary knowledge of (1) the names of the bones of the skeleton; (2) the structure and mechanism of the following joints, *i.e.*, shoulder, elbow, wrist, hip, knee, ankle; (3) the general situation of the viscera of the thorax, abdomen, and pelvis; (4) the course of the circulation of the blood; (5) the names of the various parts of the alimentary canal; (6) the principal parts of the nervous system; (7) the composition of the air; (8) the structure and general use of thermometers; (9) the signs and terms commonly used in prescriptions. They have also to pass a medical examination.

Several probationers are received for periods of not less than three months, for which the payment required is thirteen guineas a quarter, paid in advance. Their duties in the wards in no way differ from those of the ordinary probationers.

### II.—HOURS OF WORK AND TIMES OFF DUTY.

Nurses and probationers go on duty in the wards at seven a.m., leaving them in the evening at eight. They are only off duty every other day, the amount of time allowed for recreation "varying according to ward work." There are no printed time tables, so that hours are left to the individual discretion of the sisters, two hours off duty every other day being the usually accepted amount. Allowing for meals, on three or four days in each week the nurses are on duty for twelve hours at least. The special probationers have a less hard time; arriving at the hospital at half-past eight, with two hours daily in the afternoons for recreation, with extra time on Sunday, they leave the wards at eight p.m. One whole day off duty is allowed once a month. Nurses and probationers are given three weeks' holiday in the year; sisters have five weeks. Among the larger London hospitals, St. Bartholomew's shares with Guy's and the Middlesex the unenviable distinction of keeping their nurses, on certain days of the week, on duty for twelve hours and more, without a break except for brief half-hours for meals.

### III.—MEALS.

Day nurses' breakfast is at half-past six, dinner at half-past twelve, tea at half-past four, supper at nine p.m. The dietary is stated to be much the same as at most hospitals. Allowances of food are given to the nurses for each week, but not into their own keeping. It is sent from the kitchen to the ward kitchen, where a special cupboard is reserved for these provisions, which are for morning lunch, afternoon tea, and night nurses' meals. For the night meals sausages, bacon, eggs, and cake are the usual routine. A curious relic of ancient times prevails at St. Bartholomew's. The sisters provide their own breakfast, tea, and supper. Their dinner is still at the unusual hour of five.

### IV.—SALARIES AND UNIFORM.

Probationers receive in their first year £8, £12 the second, £20 the third, and £30 for the fourth. If they remain in the service of the hospital as staff nurse they receive £35 the first year, and £40 subsequently. Sisters are paid at the rate of £65 to £75. It must be remembered that out of this sum they have to partially board themselves. For uniform probationers are expected to provide their own during their trial month. If they are appointed "a certain supply of dresses, caps, and aprons" is allowed them by the hospital. "A reasonable quantity" of washing is also done for them by the hospital. Special probationers are required to provide their own uniform, with cloak and bonnet, and they also have to pay for their washing. The ordinary nurses and probationers are not required to wear outdoor uniform.

### V.—NURSES' QUARTERS.

Particulars regarding the quarters of the nurses at St. Bartholomew's Hospital are necessarily somewhat meagre, as Miss Stewart, frankly avowing that the Nurses' Home is by no means up to modern requirements, declines to allow inquirers to penetrate into its sacred precincts. It is a separate building. Want of space is the reason given for the continuance of the present condition of things, the Bluecoat School blocking the way for extensions. Probationers sleep in double-bedded rooms; staff nurses are provided with single ones. Sisters have bed and sitting rooms off their wards. The special probationers are lodged in the special probationers' home in King's Square, Goswell Road, seven-eighths of a mile from the hospital. There is no sick-room for the nurses. The sitting and dining rooms, though said to be much improved, are hardly worthy of rich St. Bartholomew's.

### Death in Our Ranks.

THE sudden death, from acute meningitis, of Miss Mary Cadbury, for six years lady superintendent of the Queen's Hospital, Birmingham, will be deeply regretted by all those amongst whom she has worked. Only a week before her death Miss Cadbury had sent in her resignation to the General Committee, on account of her health, though no serious consequences were then anticipated. Miss Cadbury began her nursing career in 1873 at the Nightingale Training Home, St. Thomas's Hospital, afterwards successively working as sister at Highgate Infirmary, district nurse in Manchester, lady superintendent of Brownlow Hill Infirmary, matron of Sheffield Public Hospital, and finally lady superintendent at Queen's Hospital from 1890. She was a member of the Royal National Pension Fund for Nurses, and of the Royal British Nurses' Association.

### Appointments.

NORWICH INFECTIOUS DISEASES HOSPITAL.—The appointment of Miss A. White as Matron was confirmed by council on September 8th. Miss White has been for five years charge nurse at the Borough Sanatorium, Brighton, and has recently acted as deputy matron at the Norwich Infectious Hospital.

### Workhouse Infirmaries and their Nurses.

By Rowland Humphreys, L.R.C.P.Lond.

The history of the rise of the workhouse and its attendant infirmary is very interesting, and might serve for the text on which to found a long article; and though the present article will be devoted to the workhouse infirmary in its hospital aspect, yet a brief retrospect will assist the reader to the comprehension of the very unsatisfactory state of things which at present exists.

So long as the monasteries existed, their revenues supported the poor and needy, the sick and infirm. With their dissolution, the State was compelled to make some provision for the same class, and, in the reign of Queen Elizabeth, overseers of the poor were appointed. These acted with the churchwardens, and later on, with the aid of inspectors, looked after the relief of the poor up to the year 1834. At this date, in consequence of the abuse of a benevolent Act which allowed almost every one to go on the rates, it was found necessary to pass a repressive Act which formed unions and governed them by the elected representatives of the ratepayers, the whole being under the supervision of a Royal Commission. In 1847 the Poor Law Board superseded the Commission, and in 1871 the duties of this Board passed into the hands of the Local Government Board, a part of the government of the country.

It will thus be seen that the present system is based on repression, a necessary consequence of the laxity of the early part of the present century.

The times have, however, greatly changed. No longer does the able-bodied labourer make the Poor Law system provide for him a place of rest and relaxation.

In 1849, according to the twenty-fourth annual report of the Local Government Board, the ratio for adult able-bodied paupers indoor and outdoor, taken together for England and Wales (excluding vagrants), was 13·2 per 1,000 of the estimated population; now (1895) it is 3·5.

For comparison sake it is to be noted that in 1858, the first year whose figures are given, the total number of not able-bodied indoor paupers was (excluding children under 16) 49,260, or about 2·5 per 1,000 of the estimated population; in 1895 the figures were 103,015, or 3·5 per 1,000; while the ratio for able-bodied indoor paupers remains about the same, thus showing the great increase in the sick department of the workhouse infirmaries. This, of course, indicates the increasing tendency of the poor to regard the workhouse infirmary in the light of a hospital for the sick.

Now what is the condition of things in these hospitals for the poor—for in many parts of the country they are the only places for treating the sick poor—and how do they compare with the metropolitan hospitals, which may be taken as a standard? Though the work in these last is very hard, and the strain on the nursing staff very great, as the expenses of such institutions are not often met by the receipts, and in consequence every economy has to be exercised.

The number of nurses of sick and insane in the poor-law infirmaries of England and Wales is 3,239. The number of sick and insane to be attended on (not including the children) amounts to 118,832, or at the rate of 38 patients to each nurse.

The *Lancet* "Metropolitan Hospital Sunday Fund Special Supplement" gives, as a typical case, one nurse to 3·4 beds (of which a certain proportion are always empty).

Thus the workhouse infirmaries, on these figures, have less than one-tenth of the number of nurses they should have to the number of patients. In this statement most of the fallacies tell against the workhouse infirmaries, for, in the first place, it is not as if all these patients were together in one perfectly-arranged building, or even series of buildings; on the contrary, they are scattered all over the country, badly arranged, without proper appliances; there are numerous wards where there should be few wards; nursing appliances are absent altogether or are very deficient; insanitary conditions, inefficient supervision, over-worked nurses all tend to make the work infinitely heavier, and with a greatly reduced general standard. If with all modern appliances, with perfect arrangements, with the most healthy conditions, one nurse to every three or four beds is necessary in order to nurse the patients properly, what can we expect from the average proportion of nurses in a poor law infirmary?

Now, to put another factor in. The greater portion of the nurses enumerated above are employed in a few well-nursed metropolitan or similar large infirmaries, and thus the results to the nursing in other infirmaries will be to diminish it to the vanishing point, to a negligable quantity. This, indeed, is the case in most of our smaller infirmaries.

The Local Government Board has recently issued one of a series of returns it is obtaining from its inspectors of workhouses. It deals with the relative proportion between nurses and patients in these institutions, and comprises Lancashire, Cumberland, and parts of Westmoreland and Yorkshire. Summarising it, it is found that in the large Lancashire workhouse infirmaries, many of them separate from the workhouse and under proper superintendence, there is only one nurse to every 14 patients; there is only one day nurse to 18, and one night nurse to 56 patients. In the smaller Lancashire infirmaries there is only one night nurse to 70 patients. Of these nurses, also, 39 per cent. have had less than a year's training, and are, therefore, unfit to be put in any position involving responsibility. This leaves each trained day nurse with 42, and each night nurse with 123 patients for whose nursing she is responsible. Including day and night nurses, there is only one nurse to 27 patients. In the Liverpool Workhouse Infirmary, to look after 1,559 patients during the night (they take very many severe cases in, and the wards are often dreadfully overcrowded), there is only one trained nurse (the superintendent), and 13 nurses who have had less than one year's training.

What chance would any voluntary hospital have of receiving subscriptions if such a state of nursing prevailed in it? Liverpool has a thoroughly competent trained infirmary matron, too, and one of the guardians is a leading authority on the nursing question. In Manchester there are 22 night nurses employed in the infirmary in charge of 1,077 patients, but 13 of these have not had a year's training. In the whole of Lancashire 20 infirmaries employ pauper nurses, people who being unable to look after themselves are employed to take care of others. Of these infirmaries, with 715 patients, 10 have no night nurse at all, and two others have no trained night nurse. Yet patients are at their worst at night, and there is no hospital medical school to take the time of nurses during the day. There is no doubt but that the employment of pauper nurses is bad from every point of view, and their employment, going as it does with absence of night nursing must, after this report, be regarded as an admission of incapacity on the part of guardians who not even know that patients require nursing at night. What healthy persons these guardians must be? One would have thought that personal experience would have taught some of them something of the needs of a sick person.

The actual state of things has been graphically told in the pages of the *British Medical Journal* by its Special Correspondent. Since these reports appeared there has been some little improvement in the attempts at trained nurses. The Local Government report, above quoted, says 62 nurses have been added in this district in 1895. The conditions under which nursing is carried on at a workhouse infirmary can, under the present system, only amount, in very many cases, to little more than attempts. The reason is not far to seek.

(*To be continued.*)

### Everybody's Opinion.

[Correspondence on all subjects is invited, but we cannot in any way be responsible for the opinions expressed by our correspondents. No communications can be entertained if the name and address of the correspondent is not given, or unless one side of the paper only be written on.]

### PROMOTION IN PROVINCIAL HOSPITALS.

A Matron writes:—"It is generally supposed by nurses who have completed a definite term of training in a good hospital that this fact alone qualifies for a sister's post. There is another fact, the recognition of which might prevent some soreness of feeling on the subject, namely, that out of, say, twenty really excellent and dear nurses there might not be one qualified by talent as well as training to fill the post and discharge the duties of a sister. Most matrons would, I think, prefer to promote her own nurses when they are possessed of the essential characteristics.

### MATRONS VERSUS MEDICAL SUPERINTENDENTS.

"Linacre" writes: The numerous investigations which have recently been made public, leads one to conclude that the whole system of management of union infirmaries must be radically wrong, emanating in the first place from the body of men who constitute "the board of guardians," who, through their lack of organisation regarding "matron's duties," apart from those of the "medical superintendent," cause, through their perhaps unintentional shortsightedness, continual unpleasantnesses and daily friction between those two upon whom the discipline of the hospital rests. In our large London hospitals we find that all the nurses are completely under the control and management of the matron, with the result that the work is carried on harmoniously. It is well known and acknowledged that a woman can manage women with greater success than a man can, and when a man in a large household, even though he be a medical superintendent, oversteps the fine line which divides the matron's province from his own, and issues orders contrary or unknown to the matron, it is then that a spirit of dissatisfaction and rebellion takes possession of the governed body—the capable and well-bred nurses resent the interference, whilst the weak and mean-spirited ones fall into the trap at the expense of their matron—and the end of it all means the matron has been rendered powerless, and her authority taken away mainly through the one whom she was engaged to "aid in the enforcement of discipline." Who is the fitter person to judge of nurses' capabilities, &c., but the matron? having been a nurse herself, and nursed amongst nurses, personal experience has taught her, and in turn fitted her to manage and control with judgment and impartiality. I hope the day will not be far distant when committees will recognise and uphold the rightful position of "the matron" in their infirmaries, and learn that the trained matron, whose services they have secured, is only in her right place when managing and controlling not only her nurses, but the whole of the female staff, whom she should have full power to engage and dismiss. Thus will her authority be strengthened amongst them, and she will become their "matron" in reality, instead of occupying the poor, miserable position she does to-day in some of our union infirmaries.

### A MATRIMONIAL OPPORTUNITY.

The following letter has reached us from an individual describing himself as "an Evangelist in connection with the Methodist Church":—

I am desirous of finding a wife who would be physically, morally, and intellectually suited for me, so that she could help me in my work, and if we had any children they might be healthy and strong. Not having succeeded in my endeavours to find the wife I require, and as people tell me that there are few who look at marriage as I do, but that the average are led into marriage by blind impulse and not by reflection and common-sense, I desire to advertise in The Hospital, and perhaps some good Christian nurse, who is strong and healthy and at the same time well educated and having the same views about marriage as I have, may be willing to devote her life with me to the improvement of the people here. If you have any doubts about my character I am willing to send references, &c., and if anyone can prove that I have any other but a pure Christian motive in advertising I am willing to forfeit a sum of £50 or £100 to any philanthropic institution you may mention. This is the kind of advertisement I would have: "An Evangelist (unsectarian), age 27, abstainer from intoxicants and narcotics, engaged in Christian philanthropic work in a small island (picturesque and healthy), is desirous of making the acquaintance of a Christian lady of about the same age, with view of marriage, if, after correspondence and medical and phrenological advice, they are convinced that they are morally, intellectually, and physically suited. She must be willing to devote her life and income (if any) for the welfare of the inhabitants, refined, well-educated, very musical, understand scientific cooking, the intellectual and moral requirements of home life, and be a thorough trained nurse; very sympathetic, gentle, with open, frank, cheerful, free nature, and very fond of children; physically strong and healthy, above average height; lungs, circulatory and digestive powers fully developed; full medium-size waist (no corset wearer), with tough and wiry body. Complexion dark or medium, hair rather dark and fine. Eyes rather dark. Full-size mouth, upper lip rather stiff; perceptive, reflective, and moral faculties fully developed. Must give proof that she is a decided Christian and abstainer, able and willing to undertake moral and intellectual work as well as nursing among the poor; that she is the daughter of healthy, intellectual, and moral parents, and that there is or has been no insanity, consumption, scrofula, &c., or anything of organic disease in the family. Apply, giving full qualifications and references. . . . ." I sign my name and address in full. I suppose some would advise me not to do so, but as I am honourable, and believe that the hereditary sufferings of humanity could be cured if only people gave up the old custom of being led by blind impulse in the choosing of a wife, I must not be afraid of being laughed at, or even misunderstood and evil spoken of.

[Our correspondent requires a good deal of his future wife, but he evidently thinks it unnecessary to state his "qualifications" as a husband. Perhaps he considers that as an evangelist his "moral and intellectual" capacities must be above suspicion, but we think the lady might justly ask what he has to offer her in return for her devotion, body and soul, to himself and his philanthropy. We only print this letter because it is an interesting example of the unconscious selfishness of the man or woman of one idea. The writer evidently thinks, with genuine self-satisfaction, that he is conferring quite a favour on the woman he seriously invites to a life of self-sacrifice and hard work, and fails to realise that women who desire to devote their lives and incomes to the service of their fellow creatures may think they can do their work far better unshackled by a husband and home cares. Should any reader of The Hospital feel inclined to "apply" for the situation, we shall be pleased to forward any letters to our correspondent.]

### Minor Appointments.

Dewsbury and District General Infirmary.—Nurse Mary Newsome has been appointed Charge Nurse at this institution. She received her training at the Royal Infirmary, Liverpool, afterwards taking up private nursing in connection with the Liverpool Nurses' Training School, Ashton Street.

Hull Sanatorium.—Miss Ada Williams and Miss M. Whilcock have been appointed Ward Sisters at this institution. Miss Williams trained in fever nursing at the Monsall Fever Hospital, remaining there for two years, and in general nursing at the Preston Royal Infirmary, where she subsequently held successively the positions of charge nurse and matron's assistant. Miss Whilcock received her training at the Sheffield General Infirmary, and at the British Lying-in Hospital, London. She held the appointment of holiday sister at the Sheffield Infirmary, and afterwards for more than two years worked on the staff of the Blackheath Nursing Institution.

### For Reading to the Sick.

### GOD'S MERCY.

**Motto.**

"He crowneth thee with loving-kindnesses and tender mercies."—*Psalm* ciii., 4.

**Verses.**

Have mercy on us, God most High,
Who lift our hearts to Thee;
Have mercy on us, worms of earth,
Most Holy Trinity.
How wonderful creation is,
The work that Thou didst bless;
And oh, what then must Thou be like,
Eternal loveliness.
—*Hymns Ancient and Modern.*

Have mercy, Lord, on me,
As Thou wert ever kind;
Let me, opprest with loads of guilt,
Thy wonted mercy find.
Wash off my foul offence,
And cleanse me from my sin;
For I confess my crime, and see
How great my guilt has been.

New mercies each returning day,
Hover around us while we pray;
New perils past, new sins forgiven,
New thoughts of God, new hopes of heaven.
If on our daily course our mind
Be set to hallow all we find,
New treasures still, of countless price,
God will provide for sacrifice.
Oh, could we learn that sacrifice,
What lights would all around us rise!
How would our hearts with wisdom talk,
Along life's dullest, dreariest walk! —*Keble.*

O Thou, that sitt'st in Heaven, and see'st
My deeds without, my thoughts within,
Be Thou my Prince, be Thou my Priest,
Command my soul, and cure my sin;
How bitter my affliction be,
I care not, so I rise to Thee.
When winter fortunes cloud the brows
Of summer friends; when eyes grow strange;
When plighted faith forgets its vows;
When earth and all things in it change,—
O Lord, Thy mercies fail me never—
When once Thou lov'st, Thou lov'st for ever.
—*F. Quarles*, 1620.

**Reading.**

"Through the tender mercy of our God, whereby the day-spring from on high hath visited us."—*S. Luke*, i., 78.

It is said that God is merciful; yes, and He is infinitely just. But can anything be more awful than when we presume upon the mercy of God to insult His justice and forbearance by transgress on His land, which is well said to be holy, just, and good; or, as our Lord has summed it up, "Thou shalt love the Lord thy God with all thy heart, and all thy mind, and all thy soul and strength; and love thy neighbour as thyself?" In the first place, we are told how our hearts may be purified, by loving an infinitely holy God, which never can be, unless we love Him and long to be holy in our hearts; which we are further directed, in the second place, to make each other happy, for His names' sake.

How great is the mercy, when we can pray with earnestness that God would take the management of the desires of the heart, that we may feel that what is the wish of our mind is the wish of our God.

"*Thoughts on Religious Subjects*," *Rev. Rowland Hill.*

### Notes and Queries.

**The contents of the Editor's Letter-box have now reached such unwieldy proportions that it has become necessary to establish a hard and fast rule regarding Answers to Correspondents. In future, all questions requiring replies will continue to be answered in this column without any fee. If an answer is required by letter, a fee of half-a-crown must be enclosed with the note containing the enquiry. We are always pleased to help our numerous correspondents to the fullest extent, and we can trust them to sympathise in the overwhelming amount of writing which makes the new rules a necessity. Every communication must be accompanied by the writer's name and address, otherwise it will receive no attention.**

**Queries.**

(169) *Expenditure Statistics.*—Being much puzzled at the omission of St. Bartholomew's and St. Thomas's Hospitals from the recent expenditure statistics, may I ask an explanation?—*M. N.*

(170) *Nursing at the Cape.*—Please tell me if you think an experienced trained nurse travelling to the Cape with an invalid brother would be likely to obtain a nursing engagement out there?—*Cape Town.*

(171) *Lunacy Certificates.*—What certificates are necessary to admit a patient (1) to a private asylum, and (2) to a county asylum?—*Superintendent.*

(172) *Male Nurse.*—Please tell me of a hospital in London where male nurses are trained. I am sixteen, and wish to undertake hospital work. —*Wolseley.*

(173) *Neuralgia.*—Can you recommend me to a specialist for this malady?—*Osseous.*

(174) *District Bags.*—Where can I get good district bags?—*A. L. M.*

(175) *Midwifery Training.*—Where can pupils obtain free training in midwifery for three or six months?—*Matron.*

(176) *Dispensing.*—Please tell me the different examinations that must be passed to become a fully qualified dispenser.—*F.*

(177) *Health Resorts.*—Please tell me the name of the book mentioned a few weeks since in The Hospital giving names of health resorts and names of English doctors in them.—*Nurse E.*

(178) *Fever Hospitals.*—What is the difference between a London fever hospital and the hospitals under the Metropolitan Asylums Board? Would you advise me to enter one for training?—*Z Z Z.*

(179) *Lectures on Nursing.*—Will you kindly tell me if there are any lectures on nursing in London which nurses from a small hospital might attend?—*Nurse.*

**Answers.**

(169) *Expenditure Statistics* (*M. N.*).—Neither St. Bartholomew's nor St. Thomas's Hospitals have been omitted in the tables given so far except in the comparative table of expenditure on meat. The reason for this is that their accounts for 1893 are not on the uniform system, and therefore do not furnish information as to the amount of meat used. For this reason it is probable that both these institutions will be omitted from future comparative tables. In any case where institutions are not mentioned it is because information has not been supplied as requested.

(170) *Nursing in South Africa* (*Cape Town*).—It is impossible to say. We always strongly dissuade nurses from venturing so far afield with no certain prospect of work, and unless they can afford a possible time of waiting. Write to the Matron of the New Somerset Hospital, Cape Town. No doubt she would be pleased to give you the benefit of her experience.

(171) *Lunacy Certificates* (*Superintendent*).—(1) In the case of private patients a petition has to be signed by the nearest relative, or by some one who must give reasons why the relative did not sign, accompanied by two medical certificates. These are taken to a Justice of the Peace, specially appointed under the Act, who grants the order of admission. Terms and conditions of admission vary so much at private asylums that it would be best to apply for particulars to the Medical Superintendent in any case. (2) Admission to county asylums is usually obtained through the relieving officer. The certificates must be signed by the relieving officer and by a Justice of the Peace, accompanied by one medical certificate. Particulars and lists of asylums, county and private, will be found in "Helps in Sickness and to Health," Scientific Press, 428, Strand, W.C.

(172) *Male Nurse* (*Wolseley*).—You are far too young to take up such work yet awhile. At present there is no male training school, properly so-called, in England, though at the National Hospital for the Paralysed and Epileptic, Queen Square, Bloomsbury, a certain number of men are trained and employed.

(173) *Neuralgia* (*Osseous*).—We cannot make any such recommendation in these columns.

(174) *District Bags.*—Write to Bailey and Sons, 38, Oxford Street, W., or to Garrould, Edgware Road, and explain what you want.

(175) *Midwifery Training* (*Matron*) *and* (*Priscilla*).—There is no such thing as free training in midwifery to be obtained. If you write to Mrs. Nichol, Midwives' Institute, 12, Buckingham Street, she will tell you where the fees are lowest, *if you enclose a stamped envelope for reply.* It is very careless and unmindful not to do this, the least return that can be made for information readily and courteously given.

(176) *Dispensing* (*F*).—Write to the Secretary, Pharmaceutical Society of Great Britain, Bloomsbury Square, W.C., from whom you can obtain full particulars.

(177) *Health Resorts* (*Nurse E.*).—B. Bradshaw's "Bathing Places and Climatic Health Resorts." (London: Kegan Paul, Trench, Trubner, and Co., Charing Cross Road, W.C.)

(178) *Fever Hospitals* (*Z Z Z.*)—The fever hospitals in London are all State supported, that is, under the Metropolitan Asylums Board, except the London Fever Hospital, Islington, to which patients are admitted by payment. Certainly it is well to gain experience in fever nursing. Write to the matrons of the various hospitals, of which you will find a complete list in Burdett's "Hospitals and Charities," Scientific Press, 428, Strand.

(179) *Lectures on Nursing* (*Nurse*).—Lectures are given by the Royal British Nurses' Association. Write to the Secretary, 17, Old Cavendish Street, for particulars. We believe a limited number of tickets may be obtained for the lectures given at the London Hospital. Write to the matron and ask.

## Figures and Tables

**Fig. 36. f1:**
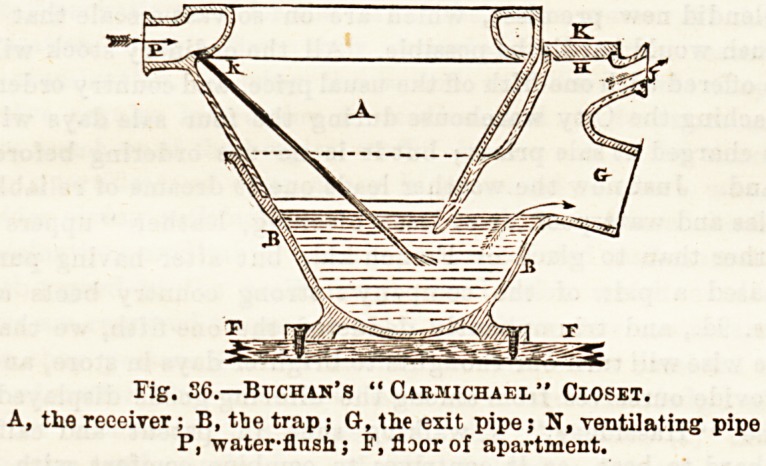


**Fig. 37. f2:**
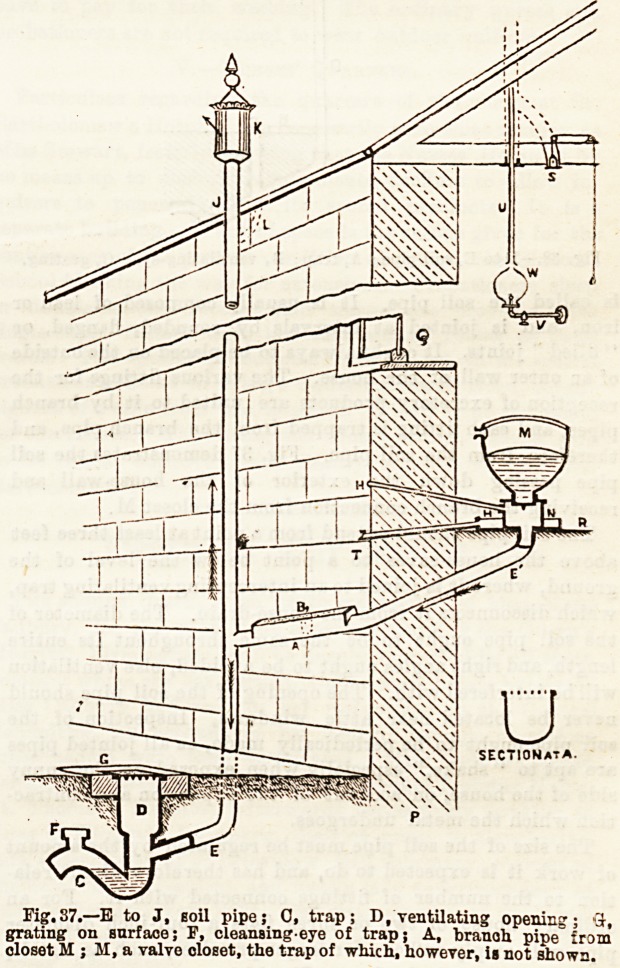


**Fig. 38. f3:**